# Targeting miR-21-3p inhibits proliferation and invasion of ovarian cancer cells

**DOI:** 10.18632/oncotarget.9216

**Published:** 2016-05-07

**Authors:** Perla M. Báez-Vega, Ileabett M. Echevarría Vargas, Fatma Valiyeva, Joel Encarnación-Rosado, Adriana Roman, Josean Flores, María J. Marcos-Martínez, Pablo E. Vivas-Mejía

**Affiliations:** ^1^ Comprehensive Cancer Center, University of Puerto Rico, Medical Sciences Campus, San Juan, Puerto Rico; ^2^ Department of Biochemistry, University of Puerto Rico, Medical Sciences Campus, San Juan, Puerto Rico; ^3^ Department of Biology, University of Puerto Rico, Rio Piedras Campus, San Juan, Puerto Rico; ^4^ Ponce Health Sciences University, Ponce, Puerto Rico; ^5^ Department of Pathology and Laboratory Medicine-University of Puerto Rico—School of Medicine, San Juan, Puerto Rico; ^6^ Puerto Rico Medical Services Administration, University of Puerto Rico, Medical Sciences Campus, San Juan, Puerto Rico

**Keywords:** ovarian cancer, microRNAs, miR-21-3p, cisplatin, RBPMS

## Abstract

MicroRNA-21 is overexpressed in most cancers and has been implicated in tumorigenesis. Accumulating evidence supports a central role for the miR-21 guide strand (miR-21-5p) in ovarian cancer initiation, progression, and chemoresistance. However, there is limited information regarding the biological role of the miR-21 passenger strand (miR-21-3p) in ovarian cancer cells. The aim of this study was to investigate the role of miR-21-3p and its target genes in cisplatin-resistant ovarian cancer cells. Expression profiling of miR-21-5p and miR-21-3p was performed in a panel of cancer cells by qPCR. Colony formation and invasion assays were carried out on ovarian and prostate cancer cells transfected with miR-21-5p and miR-21-3p inhibitors. Dual luciferase reporter assays were used to identify the miR-21-3p target genes in ovarian cancer cells. Our results show that miR-21-5p had higher expression levels compared to miR-21-3p on a panel of cancer cells. Moreover, inhibition of miR-21-5p or miR-21-3p resulted in a significant decrease in ovarian and prostate cancer cell proliferation and invasion. Luciferase reporter assays identify *RNA Binding Protein with Multiple Splicing (RBPMS), Regulator of Chromosome Condensation and POZ Domain Containing Protein 1 (RCBTB1), and Zinc Finger protein 608 (ZNF608)* as miR-21-3p target genes. SiRNA-induced RBPMS silencing reduced the sensitivity of ovarian cancer cells to cisplatin treatment. Immunohistochemical analyses of serous ovarian cancer patient samples suggest a significant decrease of RBMPS levels when compared to normal ovarian epithelium. Taken together, the data generated in this study suggests a functional role for miR-21-3p in ovarian cancer and other solid tumors.

## INTRODUCTION

MicroRNAs (miRNAs) are endogenously expressed small non-coding RNAs (nc-RNA) that regulate gene expression at the post-transcriptional level [1, 2]. MiRNAs bind to partially complementary sequences, generally in the 3′ untranslated regions (3′-UTR) of specific target mRNA molecules, leading to translation inhibition or messenger RNA degradation [1, 3]. Most miRNA genes are transcribed by RNA polymerase II into a 500-3000 bp pri-miRNAs. Pri-miRNAs are recognized and processed by the DiGeorge Syndrome Critical Region 8 (DGCR8) and Drosha proteins to generate a 70-bp hairpin loop structure-containing pre-miRNA [1, 4]. These pre-miRNAs are exported to the cytoplasm through the exportin-5 pore protein. Subsequently, they are incorporated into DICER, a complex of proteins with endoribonuclease activity that cuts away the loop joining the 3′ and 5′ arms, yielding an imperfect 22-nucleotide miRNA duplex [5]. One strand of the duplex is denoted as the miR-3p strand, and the other is the miR-5p strand [1, 6]. The duplex is incorporated into the RNA-induced silencing complex [1], where the Argonaute (Ago2) orients the mature strand (guide strand) for interaction with its target mRNAs. The other chain (passenger strand) is degraded [6]. When both strands play functional roles, the least abundant miRNA is denoted with an asterisk [5, 7].

MiR-21 (miR-21-5p), one of the best studied miRNAs, is overexpressed in most cancers and displays oncogenic activity [8]. When upregulated, miR-21-5p is implicated in all of the steps of tumorigenesis, including replicative immortalization, promotion of cell proliferation, genome instability, abnormal metabolism, angiogenesis, cell survival, invasion, metastasis, and drug resistance [5, 9–11]. Integrative genomic and massively parallel sequencing studies have shown deregulation of miR-21-5p in ovarian tumor cells [12–15]. Nam, et al. identified 23 aberrantly expressed miRNAs in ovarian cancer samples, where miR-21-5p was upregulated in 85% of the samples, as compared to normal tissue [16]. Chan, et al. demonstrated that miR-21-5p is overexpressed in cisplatin-sensitive ovarian cancer cells as compared to cisplatin-resistant cells [17]. They also reported that miR-21-5p inhibition was capable of reducing *PDCD4* expression levels and inducing apoptosis in ovarian cancer. Prior studies have shown that overexpression of miR-21-5p induces chemoresistance in several cancer types, such as breast, lung and ovarian cancer [18–20]. In addition, our group reported that upregulation of miR-21-5p through the JNK-1 pathway confers cisplatin resistance in ovarian cancer cells [21]. All accumulating evidence supports a central role for miR-21-5p and its target genes in ovarian cancer initiation, progression, and drug resistance. However, the contribution of the passenger strand (miR-21-3p) to the proliferation, invasion, and cisplatin resistance of ovarian cancer cells has not been fully elucidated. The aim of this study was to investigate the role of miR-21-3p and its target genes in ovarian cancer cells.

## RESULTS

### MiR-21-5p and miR-21-3p expression in a panel of cancer cell lines

**Figure 1:**
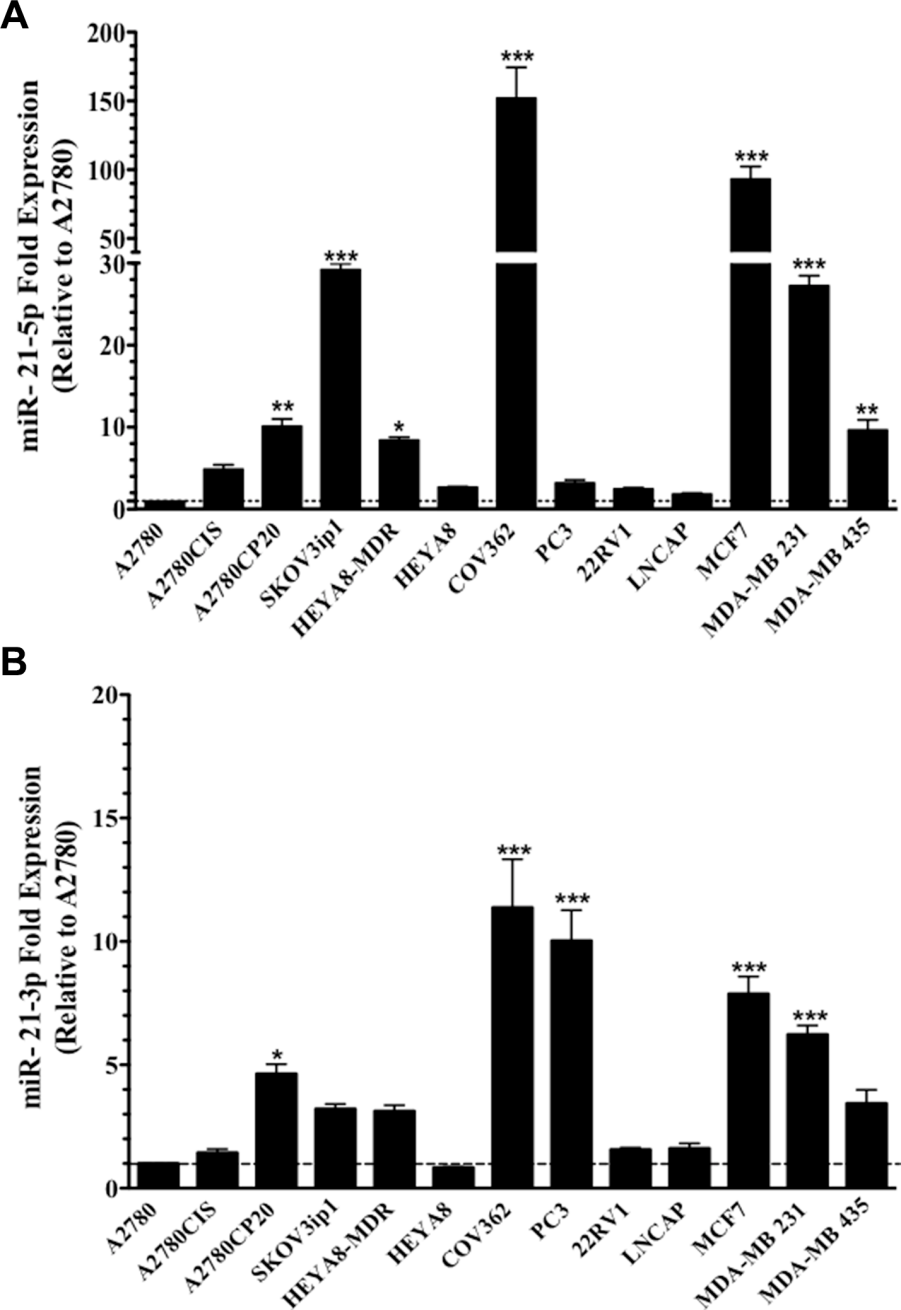
MiR-21-5p and miR-21-3p expression profiling in human cancer cell lines TaqMan-based real-time PCR analysis was performed and the threshold cycles (Ct) were used to calculate the relative (**A**) miR-21-5p and (**B**) miR-21-3p expression in cancer cell lines. Experiments were performed in triplicates. Columns represent the means ± SEM. *p ≤ 0.05, **p ≤ 0.01 and ***p ≤ 0.001.

Expression profiles of miR-21-5p and miR-21-3p were determined in a panel of human ovarian, prostate and breast cancer cells by qPCR. MiR-21-5p and miR-21-3p expression was determined by calculating relative expression levels as compared to their expression levels in the A2780 ovarian cancer cells (which expressed the lowest miR-21-5p and miR-3p expression levels). All cell lines interrogated showed higher miR-21-5p and miR-21-3p expression levels as compared with the A2780 cell line (Figure 1A–1B). The delta Ct values of miR-21-5p and miR-21-3p expression relative to the endogenous control (U44) showed that the miR-21-3p expression was lower than the miR-21-5p expression in all of the cell lines interrogated (Supplementary Figure 1).

### MiR-21-3p has a role in cell proliferation and cell invasion

**Figure 2:**
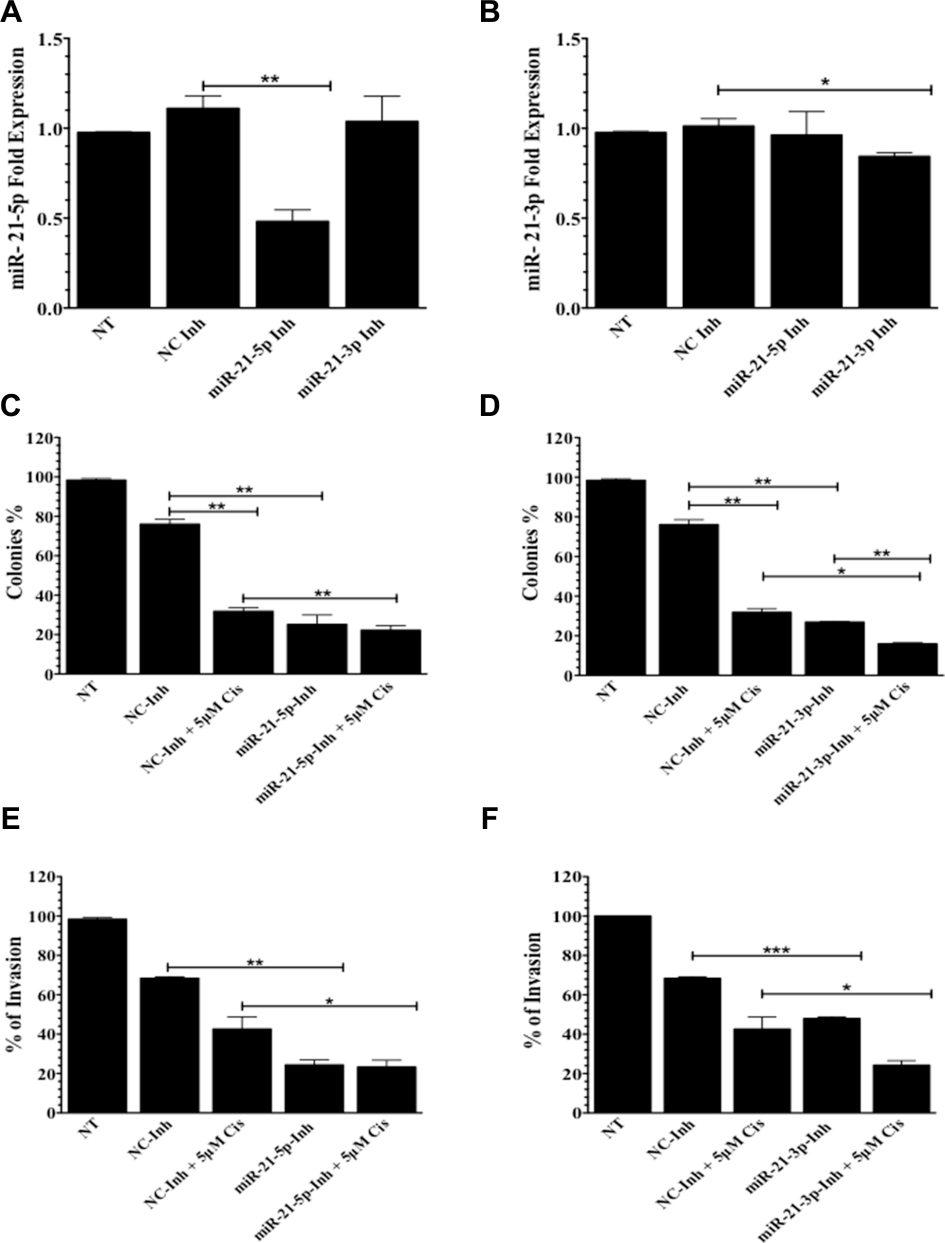
Colony formation and invasion assays in A2780CP20 cells qPCR analysis of (**A**) miR-21-5p and (**B**) miR-21-3p after transfection with miR-21-5p-Inh or miR-21-3p-Inh, respectively. Colony formation assays of (**C**) miR-21-5p-Inh or (**D**) miR-21-3p-Inh transfected cells with and without 5 μM of cisplatin. Invasion assays of (**E**) miR-21-5p-Inh or (**F**) miR-21-3p-Inh transfected cells with and without 5 μM of cisplatin. Experiments were performed at least in triplicates. Columns represent the means ± SEM. *p ≤ 0.05, **p ≤ 0.01, and ***p ≤ 0.001.

Compared to negative controls, untreated (NT) cells and a miRNA inhibitor (NC-Inh), transient transfection of A2780CP20 with specific oligonucleotide inhibitors against miR-21-5p (miR-21-5p-Inh) or miR-21-3p (miR-21-3p-Inh) significantly reduced miR-21-5p and miR-21-3p expression levels, respectively (Figure 2A–2B). MiR-21-5p expression levels decreased by 63% (**p = 0.0044) and miR-21-3p levels decreased by 17 (*p = 0.0263) compared to NC-Inh after exposure to their respective inhibitors. To determine if miR-21-5p and miR21-3p contribute to cisplatin resistance in A2780CP20 ovarian cancer cells, cell proliferation (colony formation) and invasion assays were performed in cells transfected with miR-21-5p-Inh and miR-21-3p-Inh, followed by cisplatin (5 μM, final concentration) treatment. Images of colony formation assays are shown in the Supplementary Figure 2. A2780CP20 exposed to miR-21-5p-Inh showed a significant decrease in cell proliferation compared with the NC-Inh (51%, **p = 0.0067) (Figure 2C). Cells treated with miR-21-5p-Inh and 5 μM cisplatin also exhibited decreased cell proliferation (9%, **p = 0.0047) when compared with cells transfected with NC-Inh and cisplatin (Figure 2C). Similarly, a significant decrease in cell proliferation (50%, **p = 0.0022) was observed after miR-21-3p inhibition in A2780CP20 cells when compared to NC-Inh treated cells (Figure 2D). Cisplatin treatment resulted in an additional reduction (11%, **p = 0.0067) on proliferation initiated by miR-21-3p-Inh (miR-21-3p-Inh *vs*. miR-21-3p-Inh plus cisplatin, Figure 2D). This effect was not observed with the miR-21-5p-Inh (Figure 2C).

Studies have shown that upregulation of miR-21-5p promotes cell invasion in ovarian cancer cells [21]. Therefore, we also examined if inhibiting miR-21-5p or miR-21-3p affected A2780CP20 invasive potential. Compared to NC-Inh, miR-21-5p-Inh treated cells showed a significantly reduction in cell invasion (44%, *p* = 0.0018) (Figure 2E). Similar effects were observed with miR-21-3p-Inh treatment (20%, *p* = 0.0005) (Figure 2F). Moreover, addition of cisplatin (5 μM) also reduced the number of invaded cells in miR-21-5p-Inh and miR-21-3p-Inh groups when compared to the NC-Inh and cisplatin groups (Figures 2E–2F).

**Figure 3:**
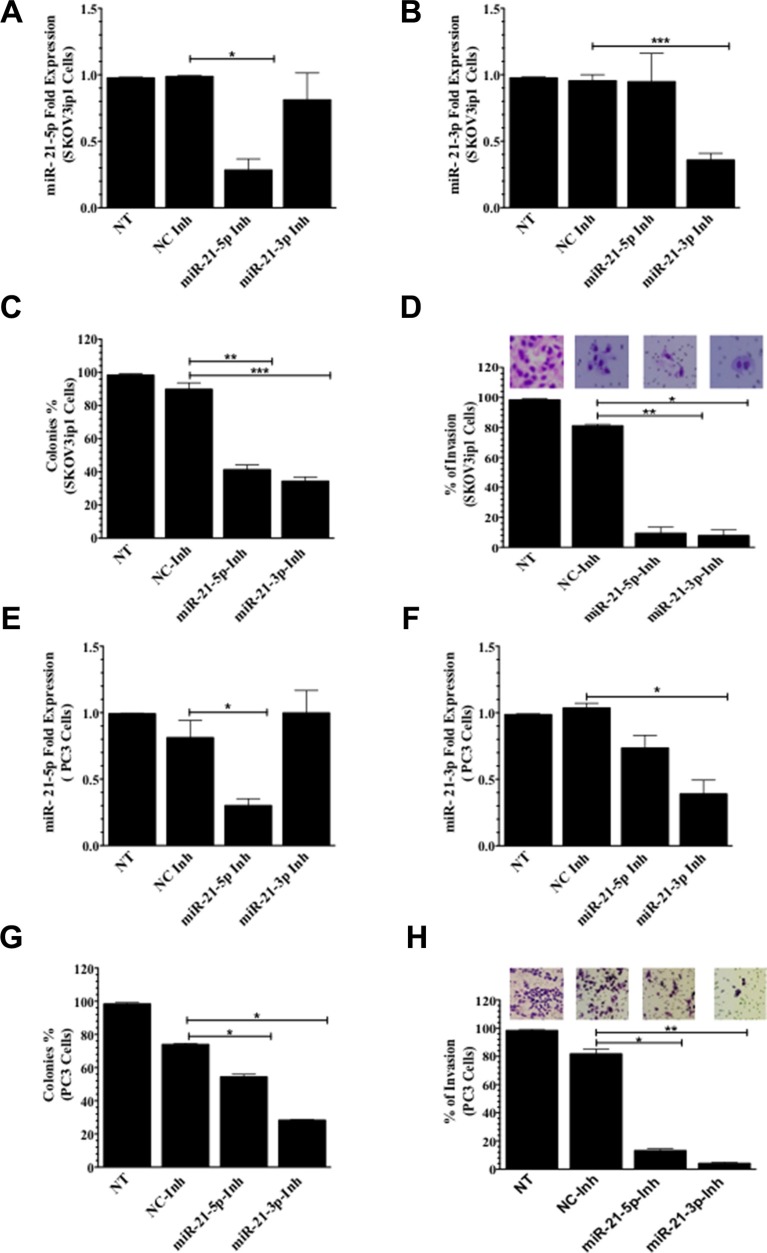
Colony formation and invasion assays in SKOV3ip1 and PC3 cells (**A**) miR-21-5p and (**B**) miR-21-3p qPCR expression following miR-21-5p-Inh and miR-21-3p-Inh transfection in SKOV3ip1 cells, respectively. (**C**) Colony formation and (**D**) invasion assays in miR-21-5p-Inh and miR-21-3p-Inh transfected SKOV3ip1 cells. (**E**) miR-21-5p and (**F**) miR-21-3p qPCR expression profiling following miR-21-5p-Inh and miR-21-3p-Inh transfection in PC3 cells, respectively. (**G**) Colony formation assays and (**H**) invasion assays in miR-21-5p-Inh and miR-21-3p-Inh transfected PC3 cells. Experiments were performed at least in triplicates. Columns represent the means ± SEM. *p ≤ 0.05, **p ≤ 0.01, and ***p ≤ 0.001.

Next, we focused on inhibiting miR-21-5p and miR-21-3p in other ovarian and prostate cancer cell lines. After transient transfection of miR-21-5p-Inh and miR-21-3p-Inh into SKOV3ip1 ovarian cancer cells, qPCR analysis showed a significant 71% (*p = 0.0373) and 57% (***p = 0.0007) decrease in miR-21-5p and mir-21-3p expression levels, respectively, when compared to NC-Inh. Mir-21-3p expression was not affected after inhibition with miR-21-5p-Inh or vice versa (Figure 3A and 3B). Inhibition of miR-21-5p and miR-21-3p in SKOV3ip1 resulted in a significant reduction in cell proliferation following miR-21-5p-Inh (48%, **p = 0.0017) and miR-21-3p-Inh (55%, ***p = 0.0007) transfection when compared to NC-Inh transfected cells (Figure 3C and Supplementary Figure 2B). Similarly, miR-21-5p-Inh or miR-21-3p-Inh significantly reduced the invasive ability of SKOV3ip1 compared to cells treated with NC-Inh (72%, **p = 0.0012, and 74%, *p = 0.0193, respectively) (Figure 2D).

To extend our study to prostate cancer models, we transfected PC3 cells with miR-21-5p-Inh. Our data showed a significantly decrease 51% (*p = 0.0311) in miR-21-5p expression levels when compared to NC-Inh (Figure 3E). Similarly, inhibition with miR-21-3p-Inh showed a significant 64% decrease (***p = 0.045) in miR-21-3p expression levels (Figure 3F). Mir-21-3p expression was not affected after inhibition with miR-21-5p-Inh and vice versa. Inhibition of miR-21-5p or miR-21-3p in PC3 cells significantly reduced the number of cell colonies by 20% (*p = 0.0359) and 45% (*p = 0.0198), respectively (Figure 3G and Supplementary Figure 2C). Transfection of miR-21-5p-Inh or miR-21-3p-Inh reduced the number of invaded PC3 cells by 69% (*p = 0.0327) and 78%, (**p ≤ 0.0024), respectively, when compared to cells transfected with NC-Inh (Figure 3H).

### Computational analysis predicts RBPMS, RCBTB1 and ZNF608 as miR-21-3p target genes

**Figure 4:**
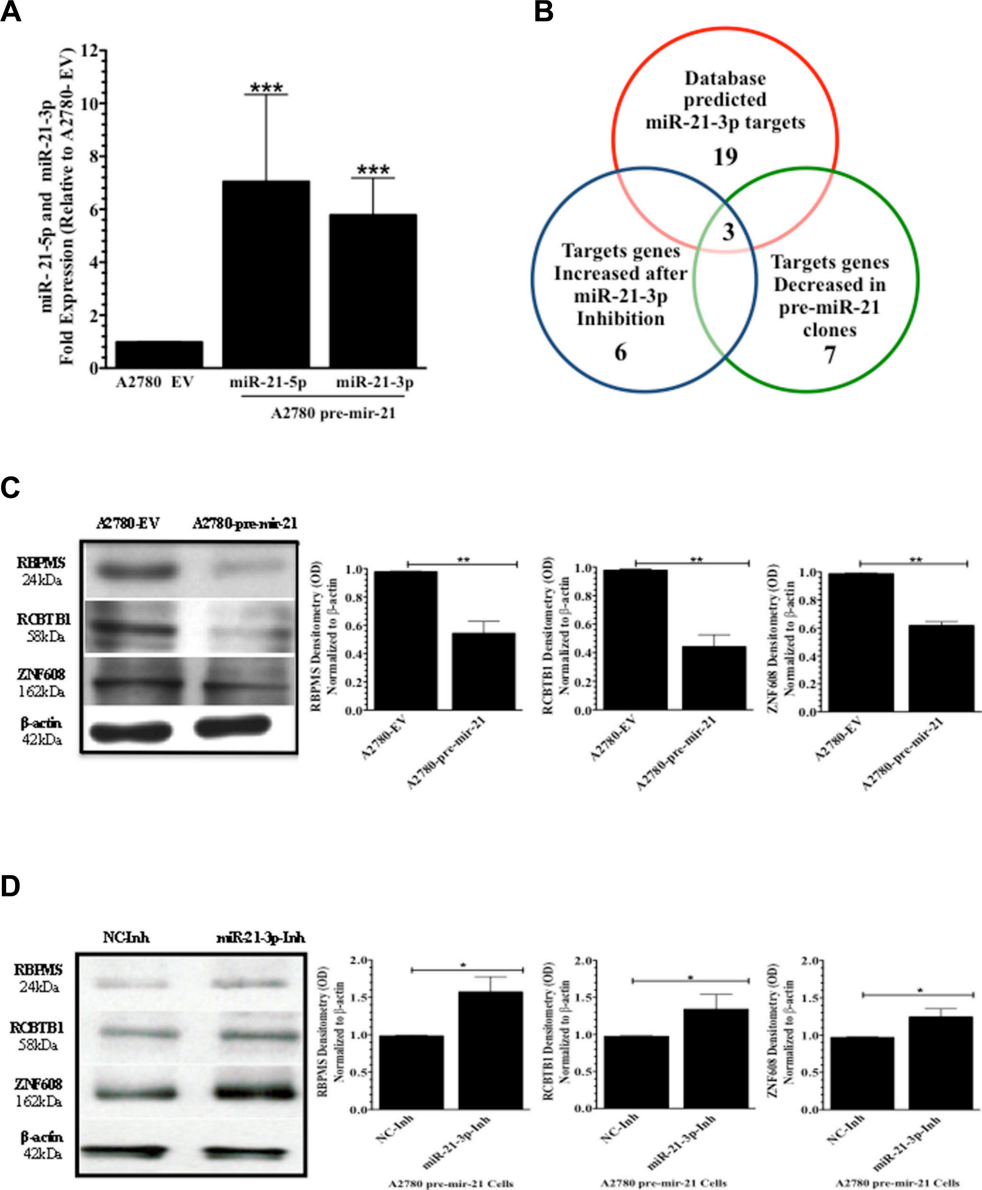
MiR-21-3p target identification (**A**) qPCR expression analysis of miR-21-5p and miR-21-3p in A2780-EV (empty vector) and A2780 pre-mir-21 clones. (**B**) Venn diagram showing the potential miR-21-3p regulated genes in A2780CP20 cells. (**C**) Western blots and densitometric analysis of the band intensities of RBPMS, RCTB1, and ZNF608 protein levels in A2780 pre-mir-21 cells compared to A2780 EV cells. β-actin was used for normalization. (**D**) Western blots and densitometry analysis of the band intensities for RBPMS, RCTB1, and ZNF608 in A2780 pre-mir-21 cells transfected with miR-21-3p Inh or NC-Inh. Experiments were performed at least in triplicates. Columns represent the means ± SEM. *p ≤ 0.05, **p ≤ 0.01, ***p ≤ 0.001, and ****p <0.0001.

qPCR analysis showed that the A2780 pre-mir-21 clone used in this study had a 9.8-fold (****p < 0.0001) and a 5.7-fold (****p < 0.0001) increase in miR-21-5p and miR-21-3p expression, respectively, compared to the A2780-EV (empty vector) clone (Figure 4A). Using the publicly available miRBase software (accesses the following databases: miRDB, microRNA.org, and Diana Lab) three different software programs predicted 19 potential miR-21-3p target genes. Supplementary Table 1 includes miR-21-3p-specific binding sites in each of the 19 predicted target genes.

Using total RNA isolated from A2780-pre-mir-21 or A2780-EV clones, qPCR analysis was performed to compare relative expression levels of all predicted miR-21-3p target genes (Supplementary Figure 3A). Seven out of 19 genes had decreased expression in A2780-pre-mir-21 clones compared to A2780-EV clones (Table 1). These genes included to *ZNF608*, *ZFHX3*, *DAB2IP*, *RBPMS*, *ZNF217*, *RCBTB1* and *SMAD7* (Table 1). Next, qPCR was performed with total RNA isolated of A2780-pre-mir-21 clones transiently transfected with miR-21-3p-Inh or NC-Inh. Only three genes (*RBPMS*, *RCBTB1 and ZNF608*) had increased expression in A2780CP20 miR-21-3p-Inh transfected cells compared to NC-Inh transfected cells (Table 1 and Supplementary Figure 3B). Computational analyses and qPCR gene expression profiling results are summarized in the Venn diagram depicted the Figure 4B.

**Table 1:** List of the 19 miR-21-3p predicted target genes using *in silico* analysis, and qPCR results

1	2	3	4	5
Accession Number	Full name	Gene Symbol	Fold change A2780-pre-miR21 *vs* A2780-EV	Fold change miR-21-3p-Inh *vs* NC-miR Inh
NG_029918.1	BTG3 Associated Nuclear Protein	BANP	0.9476226	1.378739
NG_032815.1	Lin-28 Homolog B (C. elegans)	LIN28B	1.155845	0.6929104
NG_012641.1	Calcium Channel, Voltage-Dependent, Beta 4 Subunit	CACNB4	9.646072	2.755901
NG_008722.1	Lysyl Oxidase	LOX	37.566060	2.184768
NM_031442.3	Transmembrane Protein 47	TMEM47	238.482500	0.009252672
NM_001282959.1	Cell Division Cycle and Apoptosis Regulator 1	CCAR1	2.666675	0.2038651
NG_016776.1	Melanoma Antigen Family L2	MAGEL2	10.848550	29.626190
NM_020747.2	Zinc Finger Protein 608	ZNF608	0.1578171	1.380395
NM_001145306.1	Cyclin-Dependent Kinase 6	CDK6	4.178234	9.645449
NM_007217.3	Programmed Cell Death 10	PDCD10	3.965824	2.170048
NM_001164766.1	Zinc Finger Homeobox 3	ZFHX3	0.4123704	0.5130655
NM_032552.3	DAB2 Interacting Protein	DAB2IP	0.3358732	0.1714943
NM_001008710.2	RNA Binding Protein with Multiple Splicing	RBPMS	0.8511509	2.710083
NM_006526.2	Zinc Finger Protein 217	ZNF217	0.770239	0.7251549
NM_203394.2	E2F Transcription Factor 7	E2F7	1.204480	0.2603122
NM_018191.3	Regulator of Chromosome Condensation (RCC1) and BTB (POZ) Domain Containing Protein 1	RCBTB1	0.4420137	1.866371
NM_001190274.1	F-box Protein 11	FBXO11	1.352711	3.812189
NM_001190821.1	SMAD family member 7	SMAD7	0.2718375	0.3114765
NM_032288.6	Forty-two-three domain containing 1	FYTTD1	1.433740	2.398536

Western blot analysis showed that RBPMS, RCBTB1, and ZNF608 protein levels were downregulated in A2780 pre-mir-21 clones compared to A2780-EV clones (Figure 4C). Densitometry analysis showed a significant decrease (**p ≤ 0.01) in RBPMS, RCBTB1, and ZNF608 protein levels in A2780-pre-miR-21 clones compared to A2780-EV cells (Figure 4C). Transient transfection of the miR-21-3p inhibitor into A2780 pre-mir-21 clones reduced RBPMS, RCBTB1, and ZNF608 protein levels compared to clones transfected with the NC-Inh (Figure 4D). Densitometry analysis of the band intensity showed a significant increase (*p ≤ 0.05) in RBPMS, RCBTB1, and ZNF608 protein levels in A2780-pre-mir-21 clones transfected with miR-21-3p-Inh compared to clones transfected with the NC-Inh (Figure 4D). Table 2 shows the biological role of these three miR-21-3p potential target genes.

**Table 2:** Biological role of the three potential miR-21-3p target genes

Accession Number	Full Name	Biological Role
NM_018191.3	Regulator of Chromosome Condensation (RCC1) and BTB (POZ) Domain: RCBTB1	May be involved in cell cycle regulation by chromatin remodeling. In humans, this gene maps to a region of chromosome 13q that is frequently deleted in B-cell chronic lymphocytic leukemia and other lymphoid malignancies [44].
NM_001008710	RNA binding protein with multiple splicing: RBPMS	Acts as a coactivator of transcriptional activity. Required to increase TGFB1/Smad-mediated transactivation. Acts through SMAD2, SMAD3 and SMAD4 to increased transcriptional activity. Promotes the nuclear accumulation of SMAD2, SMAD3 and SMAD4 proteins [42].
NM_020747.2	Zinc Finger Protein 608: ZNF608	Putative transcriptional repressor regulating G2/M transition. Involve in transcription, cell division and chromosome partitioning [5].

### MiR-21-3p binds to RBPMS, RCBTB1, and ZNF608 3′-UTR regions in ovarian cancer cells

**Figure 5:**
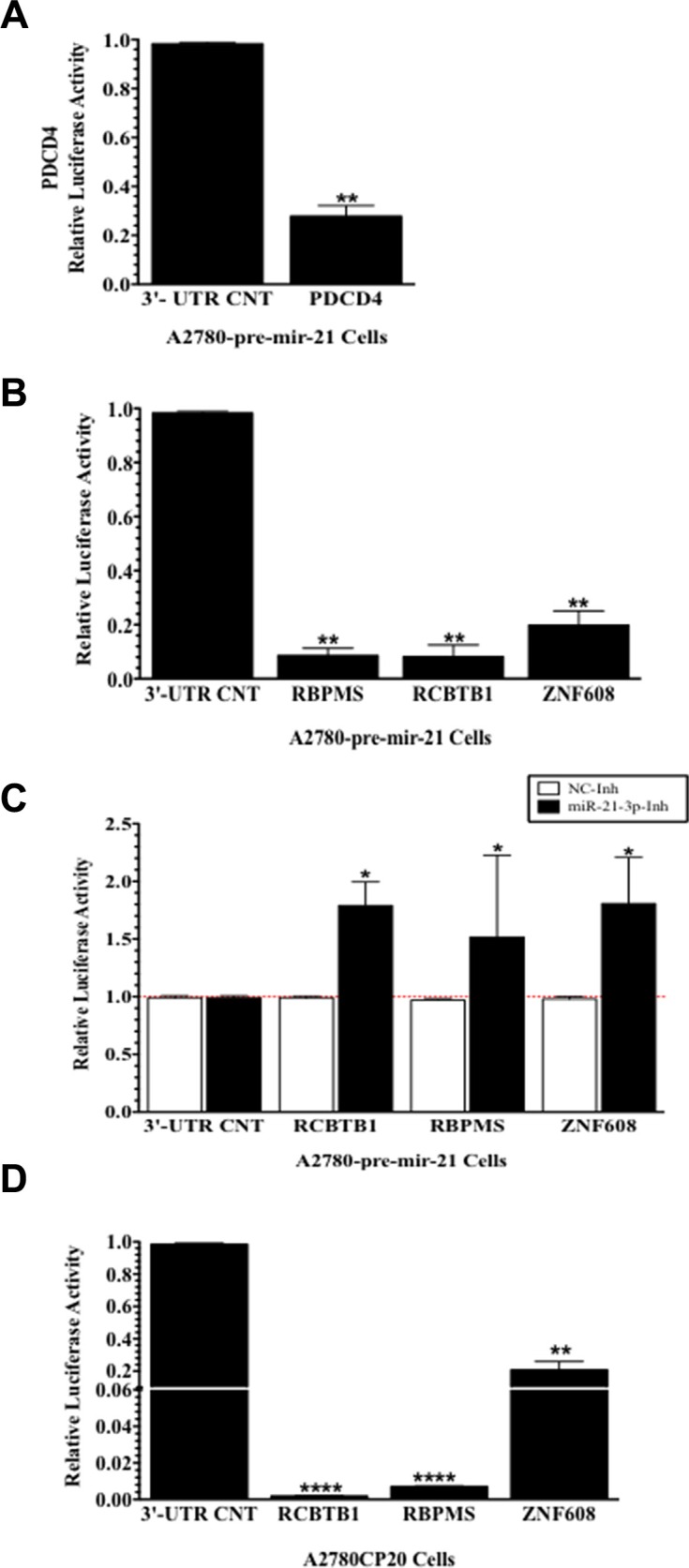
Validation of miR-21-3p targets by luciferase reporter assays (**A**) Relative luciferase activity in A2780 pre-mir-21 cells transiently transfected with PDCD4 and negative control 3′-UTR vectors. (**B**) Relative luciferase activity in A2780 pre-mir-21 cells transiently transfected with a *RBPMS, RCBTB1, ZNF608* or negative control 3′-UTRs vecttors. (**C**) Relative luciferase activity in A2780 pre-mir-21 cells transiently co-transfected with the mir-21-3p-Inh and the *RBPMS, RCBTB1, ZNF608* or negative control 3′-UTR vectors. (**D**) Relative luciferase activity in A2780CP20 cells transiently transfected with a *RBPMS, RCBTB1, ZNF608* or negative control 3′-UTRs vectors. Experiments were performed at least in triplicates Columns represent the means ± SEM. *p ≤ 0.05, **p ≤ 0.01, and ***p ≤ 0.001.

To directly confirm that miR-21-3p binds to the 3′-UTR of *RBPMS, RCBTB1*, and *ZNF608*, dual luciferase reporter assays were performed. A2780 pre-mir-21 clones were transiently transfected with luciferase vectors containing the 3′-UTR of *RBPMS, RCBTB1*, *ZNF608*, or a 3′-UTR control (3′-UTR CNT). As a positive control, we transiently transfected the PDCD4 (Programmed Cell Death 4) 3′-UTR vector in the A2780 pre-mir-21 cells. As expected, relative luciferase activity of PDC4 3′-UTR was significantly decreased (73%, **p ≤ 0.0001) when compared to 3′-UTR control (Figure 5A). Transfection of *RBPMS, RCBTB1*, and *ZNF608* 3′-UTR vectors showed a significant reduction (91.5%, **p ≤ 0.0028, 92%, **p ≤ 0.0039 and 80%, **p = 0.0039, respectively) in relative luciferase activity compared to the 3′-UTR control vector (Figure 5B). To confirm that RBPMS, RCBTB1, and *ZNF608* are miR-21-3p targets, we co-transfected the A2780-pre-mir-21 cells with the miR-21-3p inhibitor (miR-21-3p-Inh) or the negative control inhibitor (NC-Inh), and the 3′-UTR vectors of *RBPMS, RCBTB1*, or ZNF608. MiR-21-3p inhibition followed by transfection of the 3′-UTR vectors of *RBPMS, RCBTB1*, or ZNF608 resulted in a significant increase (*p ≤ 0.05) in the relative luciferase activity of the three targets tested when compared to cells co-transfected with the NC-Inh (Figure 5C). In A2780CP20 cells, transiently transfection of the 3′-UTR vectors for *RBPMS, RCBTB1*, or *ZNF608*, resulted in a significant decrease of the relative luciferase activity for the three targets (RBPMS: 97%, ****p < 0.0001; RCBTB1: 98%, ****p < 0.0001; and ZNF608:20%, **p ≤ 0.0001) when compared to the 3′-UTR CNT (Figure 5D).

### Effects of RBPMS, RCBTB1, and ZNF608 silencing on the sensitivity of ovarian cancer cells to cisplatin treatment

**Figure 6:**
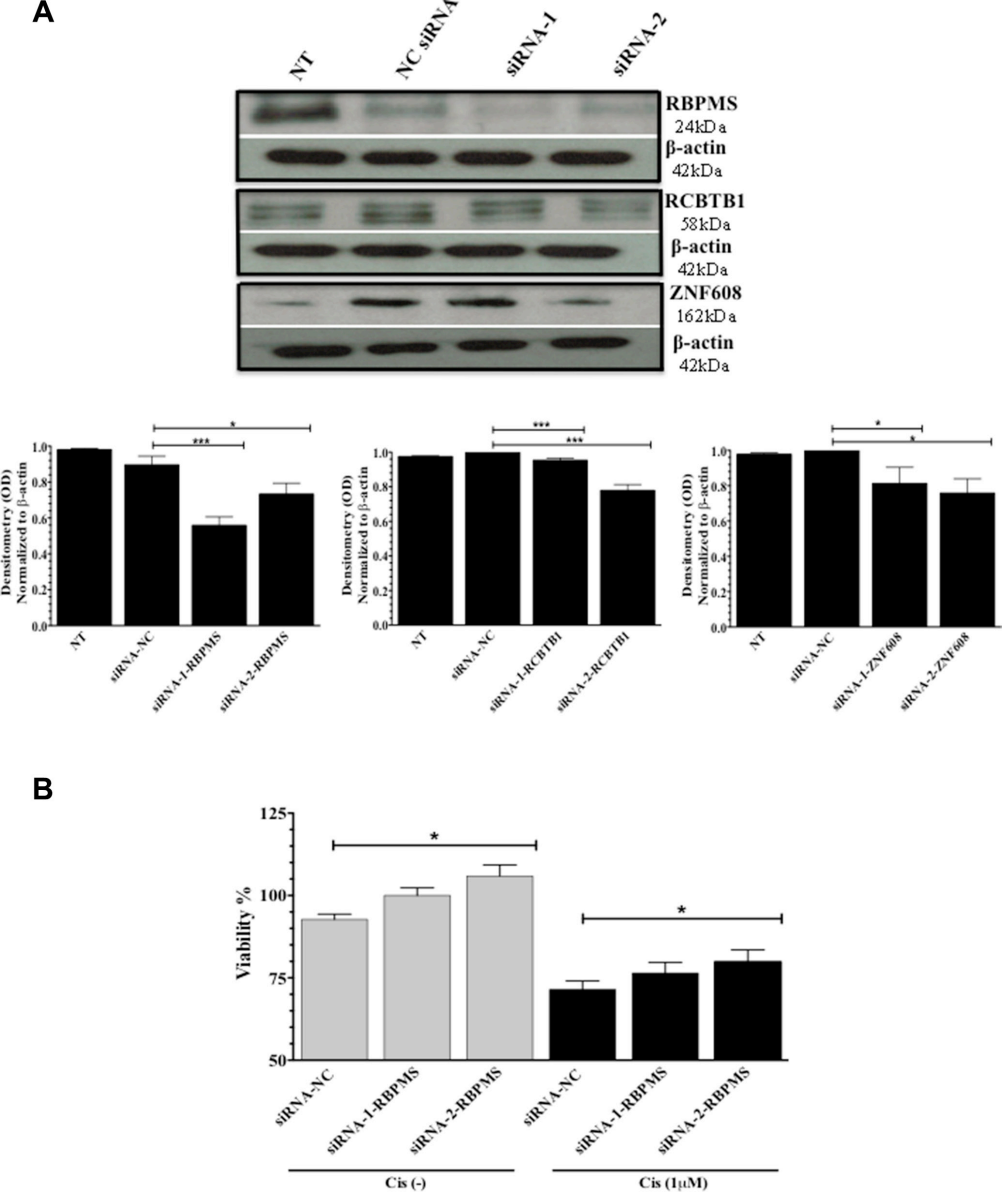
SiRNA-mediated gene silencing A2780 cells were transfected with 2 siRNAs for each: RBPMS, RCBTB1 or ZNF608 of with a NC-siRNA. (**A**) Western blots and densitometric analysis of the band intensities of RBPMS, RCBTB1, and ZNF608 protein levels after silencing with the siRNAs. β-actin was used for normalization. (**B**) Percentage of viable A2780 cells after transfection with siRNA-1-RBPMS or siRNA-2-RBPMS, in the presence or absence of cisplatin (Cis) (1 μM final concentration). Experiments were performed in triplicates. Columns represent the means ± SEM. *p ≤ 0.05, **p ≤ 0.01, and ***p ≤ 0.001.

A2780 ovarian cancer cells expressed lower levels of miR-21-3p and higher levels of RBPMS, RCBTB1, and ZNF608 as compared to A2780CP20 cells. Thus, we examined whether the downregulation of RBPMS, RCBTB1 and ZNF608 protein expression levels in A2780 reduced the sensitivity of these cells to cisplatin treatment. Two siRNAs targeting different mRNA regions of each gene were designed. Western blot analyses confirmed that two siRNA against RBPMS, RCBTB1, or ZNF608 did significantly reduced their protein levels (Figure 6A). Densitometric analysis of bands intensity confirmed these findings (Figure 6A). SiRNA-mediated gene silencing (50 nM final concentration) of RCBTB1 or ZNF608 followed by cisplatin treatment (1 μM) did not have a significant effect on A2780 cell viability (Supplementary Figure 4A). However, silencing of RBMPS significantly increased the cell viability by 15% (*p = 0.0160) compared to the negative control (NC)-transfected cells (Figure 6B). Addition of 1 μM cisplatin (48-hr treatment) reduced the sensitivity of A2780 cells to cisplatin treatment, as evidenced by the increase in cell viability (8%, *p = 0.0175) of siRNA-2-RBPMS and cisplatin combination compared with the NC-siRNA and cisplatin combination group (Figure 6B).

### RBPMS protein expression in normal ovaries and serous ovarian cancer

**Figure 7:**
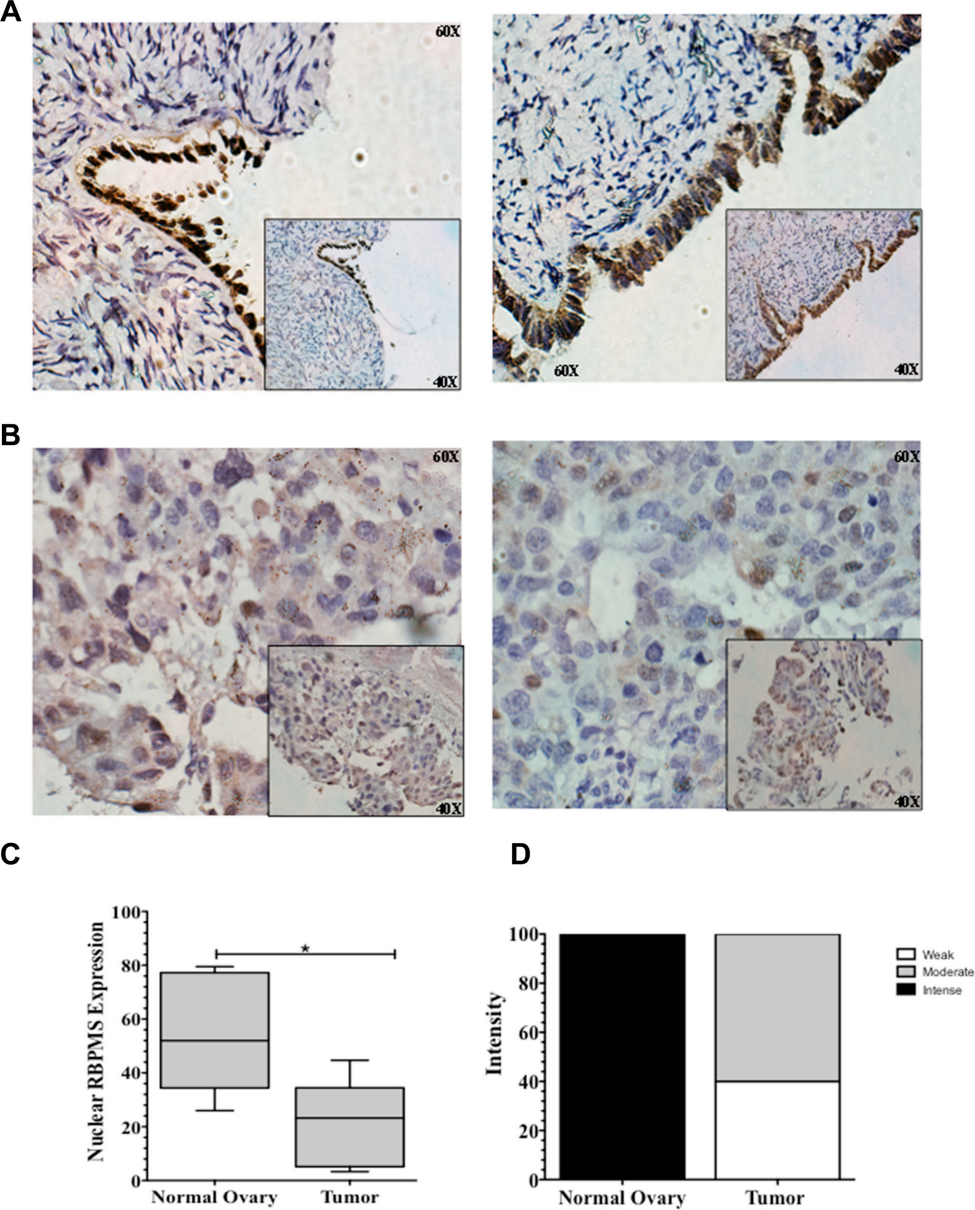
RBPMS expression in serous ovarian cancer samples Representative images of RBPMS immunostaining of (**A**) two normal ovary, and (**B**) two ovarian cancer patients. Magnification: 60×. Insert 40×. (**C**) Box plots showing the nuclear RBPMS immunostaining levels in normal ovary and serous ovarian cancer tissues. A line cross the box indicates the median value. Error bars in the box represent the means ± SEM. *p ≤ 0.05. (**D**) RBPMS immunostaining intensity was found to be weaker in serous ovarian tumor specimens when compared to normal ovaries.

To further determine potential clinical relevance in ovarian cancer, RBPMS protein expression patterns were examined in formalin fixed paraffin embedded (FFPE) samples by immunohistochemical analysis (IHC). The characteristics of the patients are described in Table 3. Nuclear positive RBPMS staining at the ovarian surface epithelial cells was observed in all control samples examined as shown in the representative IHC images in Figure 7A. On the other hand, most ovarian cancer epithelial cells showed moderate or negligible RBPMS staining (Figure 7B). Statistical analysis of the IHC results revealed RBPMS expression to be significantly higher (*p = 0.0179) in epithelial cells of normal ovaries as compared to tumor tissue (Figure 7C). Immunohistochemical intensity of RBPMS was higher in normal ovaries as compared to tumor tissue (Figure 7D).

**Table 3:** General characteristics of normal ovary and serous ovarian cancer tissues

	No. of Cases	Mean Age ± SD	Histologic Grade
Normal Ovary	5	51.89 ± 8.95	–
Serous Ovarian Cancer	5	58.80 ± 11.63	High Grade

## DISCUSSION

The role of miR-21 (miR-21-5p) has been amply studied in several types of cancer, and it has been proposed as an oncogene [8]. Aberrantly increased levels of miR-21-5p have been associated with cancer initiation, progression, and metastasis [5]. In ovarian cancer, increased levels of miR-21-5p have also been associated with cisplatin resistance [20, 21]. However, the role of the miR-21 “passenger” strand (miR-21-3p) in the proliferation, invasion, and drug resistance of ovarian cancer cells has not been fully elucidated. The aim of this study was to investigate the role of miR-21-3p and its target genes in drug resistant ovarian cancer cells. Our findings support that miR-21-3p, the “passenger” strand of pre-miR-21, has an important role in cell proliferation, invasion, and drug resistance of ovarian cancer cells. In addition, we identified *RBPMS*, *RCBTB1* and *ZNF608* as targets of miR-21-3p in cisplatin-resistant ovarian cancer cells.

Although miR-21-3p expression was lower compared to miR-21-5p expression in all cell lines tested, our findings are in agreement with previous evidence suggesting that the passenger strand is usually less abundant than the mature miRNA strand [22]. Inhibition of miR-21-5p and miR-21-3p in A2780CP20 ovarian cancer cells showed a significant decrease in cell proliferation and cell invasion. Similar effects were observed when inhibiting miR-21-5p and miR-21-3p in SKOV3ip1 ovarian cancer and PC3 prostate cancer cell lines. Together, this data confirm previous findings showing that very low expression levels of particular genes could have significant biological and molecular downstream effects [23]. In fact, targeting miR-21-3p had a more pronounced effect in the inhibition of the invasion ability of SKOV31p1 and PC3 than inhibiting the miR-21-5p in these cell lines. These results suggest that miR-21-3p and its target genes could have important biological roles in the carcinogenesis and drug resistance of other solid tumors. This is not the first time that two complementary strands of a pre-miRNA have been shown to have biological roles. Kuchenbau et al. showed that the miR-223 duplex acts through both arms in myeloid cells [24]. Both, miR-223 and miR-223* target the insulin-like growth factor 1 receptor, and high miR-223* levels were associated with increased overall survival in patients with acute myeloid leukemia [24]. Similarly, miR-28-5p and miR-28-3p were found to be down-regulated in colorectal cancer (CRC) [25]. Overexpression of each miRNA strand in CRC had different biological effects. Overexpression of miR-28-5p reduced CRC cell proliferation, migration, and invasion *in vitro*, whereas overexpression of miR-28-3p increased CRC cell migration and invasion *in vitro* [25].

Combination treatment with miR-21-5p or miR-21-3p inhibitors, and cisplatin showed significant decreases in cell proliferation and cell invasion. Prior reports have shown that upregulation of miR-21-5p contributes to cisplatin resistance in ovarian cancer cells [20, 21]. Recently, Pink et al. showed that miR-21-3p increased resistance to cisplatin in a range of ovarian cell lines [20]. They identified Neuron Navigator 3 (NAV3) as a potential miR-21-3p target. RNA-seq analysis of the 284 ovarian cancer samples available in “The Cancer Genome Atlas” (TCGA) data portal showed a significant correlation between miR-21-3p overexpression, NAV3 downregulation, and cisplatin resistance [20]. These results suggest that both miR-21-3p and miR-21-5p could contribute to the cell growth, proliferation, invasion and the cisplatin resistance of ovarian cancer cells.

Of the 19 predicted miR-21-3p target genes analyzed, only three genes (*RBPMS*, *RCBTB1 and ZNF608*) were confirmed as miR-21-3p target genes in ovarian cancer cells. RBPMS (RNA-binding protein with multiple splicing) mediates the transcriptional activity of SMAD proteins, mainly by enhancing the phosphorylation of SMAD2 and SMAD3 [26]. Upon phosphorylation, SMADs accumulate in the cell nucleus and act as mediators of transcriptional activation [26]. RCBTB1 (RCC1 and BTB domain-containing protein 1) has been associated with critical cellular processes including cell cycle and transcriptional control through chromatin remodeling [5]. The ZNF608 (zinc finger protein), also known as renal carcinoma antigen NY-REN-36, is thought to be involved in transcriptional regulation events [27]. We observed that the reduced expression of RBPMS increased cell growth and reduced sensitivity of ovarian cancer cells to cisplatin treatment. These findings suggest that RBPMS is a relevant miR-21-3p target gene in ovarian cancer cells. Recently, Fu, et al. reported that RBPMS inhibited proliferation and migration of breast cancer cells by blocking the formation of c-Jun-c-Fos or c-Jun-Smad3 complexes [28]. Further studies are needed to clarify the biological role and the clinical significance of RBPMS in ovarian cancer.

As for the human data, we acknowledge that the number of ovarian cancer samples analyzed by IHC is not optimal to reach a definite conclusion on the role of RBPMS in ovarian cancer. However, our data supports the IHC data published in the human protein atlas data portal (http://www.proteinatlas.org/cancer) showing negative/low RBPMS staining in their database that includes 12 human ovarian cancer samples. Future studies are needed to confirm the RBPMS protein expression patterns in ovarian cancer patients.

In summary, we showed that inhibiting miR-21-3p in ovarian and prostate cancer cells induced similar effects, or even more pronounced effects, than the inhibition of miR-21-5p. Although the role of miR-21-3p target genes in ovarian cancer remains to be elucidated, prior reports and our findings support the hypothesis that both strands of the pre-mir-21, miR-21-5p and miR-21-3p, promote cancer growth by regulating their own target genes. Further studies are needed to investigate the biological role of the miR-21-3p target genes in ovarian cancer and other solid tumors.

## MATERIALS AND METHODS

### Cells culture

The human ovarian epithelial cancer cells lines A2780CP20, SKOV3ip1, HEYA8 and HEYA8.MDR were generous gifts from Dr. Anil K. Sood from MD Anderson Cancer Center and have been previously described elsewhere [29–34]. The prostate cancer cell lines PC3, 22RV1, and LNCAP and the breast cancer cell lines MCF7, MDA-MB 435 (reclassified as Melanocyte, Melanoma), and MDA-MB 231 were purchased from the American Type of Culture Collections (ATCC) at Manassas, VA, USA. All of these cell lines were obtained in 2010 and authenticated in 2013 by Promega and the ATCC using short tandem repeat (STR) analysis. A2780 and A2780CIS cells were purchased in 2012 from the European Collection of Cell Cultures (ECACC) at Salisbury, UK where authenticated cell lines are provided. The cells were propagated *in vitro* in RPMI-1640 medium supplemented with 10% fetal bovine serum (FBS) and 100 U/mL penicillin (Thermo Scientific), and 100 μg/mL streptomycin (Thermo Scientific). Cells were maintained in a humidifier chamber at 37°C with 5% CO_2_. Before use, all tumor cell lines were incubated with Mycoplasm Removal Agent (AbD Sertotec, NC, USA) as a preventative measure described by the manufacturer. *In vitro* assays were performed at 70–85% cell density.

### RNA isolation and cDNA synthesis

Total RNA (including microRNAs) was isolated using the mirVana microRNA Isolation Kit (Life Technology, Grand Island, NY). RNA concentrations were read using a Nanodrop 2000 (Thermo Scientific, Waltham, MA, USA). Total RNA was converted into cDNA with the TaqMan MicroRNA Reverse Transcription Kit according to manufacturer's instructions (Life Technology, Grand Island, NY). Subsequently, cDNAs were stored at −80°C.

### Real time PCR (qPCR)

The cDNAs were amplified using specific TaqMan MicroRNA Assays (Life Technology, Grand Island, NY) for miR-21-5p (hsa-miR-21-5p, probe number 000397) and miR-21-3p (hsa-miR-21-3p, probe number 002438). The RNU44 TaqMan MicroRNA Assay was used as an endogenous control (EC). Briefly, each qPCR reaction was carried out in a total volume of 10 μl containing 1 μl of 20X TaqMan microRNA assay, 1.33 μl of TaqMan 2X universal PCR master mix, and 7.67 μl of nuclease free water. The following qPCR conditions were used: 10 minutes at 95°C followed by 40 cycles of 15 seconds at 95°C, and 60 seconds at 60°C. Data was collected and analyzed using the StepOne software v2.1 (Life Technology, Grand Island, NY). The threshold cycle was used to calculate relative miRNA expression levels as previously described [35].

### Transient and stable transfections

Ectopic miR-21 expression was performed in A2780 cells. pCMV-MIR21 or an empty vector (pCMV-EV) (OriGene Technologies, Inc. Rockville, MD) was transfected into A2780 cells. After 2–3 weeks, independent colonies were picked and cultured separately as independent clones [21]. To inhibit miR-21-5p or miR-21-3p expression, we transiently transfected A2780CP20, SKOV3ip1, and PC3 cells with a miR-21-5p or a miR-21-3p oligonucleotide inhibitor (Life Technologies). A negative oligonucleotide inhibitor was used as a control (Life Technology, Carlsbad, CA). MiRNA inhibitors were always mixed with the Lipofectamine 2000 transfection reagent (Life Technologies) and Opti-MEM I growth media (Life Technologies). Eight hours after transfection, the culture media was replaced by RPMI (10% FBS) and cells were collected 24 hours after transfection.

### Colony formation assays

Cells (3.5 × 10^4^ cells/ml) were seeded in six well plates. After 24 hours, the transfection reaction mix containing Opti-MEM I (without FBS and antibiotics), 50 nM (final concentration) of a miR-21-5p inhibitor, a miR-21-3p inhibitor, or a negative control-miRNA inhibitor (NC-miR), and Lipofectamine 2000 transfection reagent in a 1:4 ratio was added to the cells. Eight hours post-transfection, the culture media was removed and replaced by regular RPMI media (with 10% FBS). Twenty-four hours post-transfection, 1,000 cells were seeded in 10 cm Petri dishes. Ten-12 days later, colonies were stained with 0.5% crystal violet in methanol. Colonies of at least 50 cells were counted in five random fields (10×) using the Nikon eclipse TS100 microscope. The percentage of colonies was calculated relative to the number of colonies in the control miRNA inhibitor plate, which was considered as 100%.

### *In vitro* invasion assay

Cells (3.5 × 10^4^ cells/ml) were plated in Petri dishes and transfected with miRNA inhibitors as described for the colony formation assays. The next day, 60 μl of diluted matrigel (serum-free RPMI media) was added into upper chamber of 24-well transwell plate (Corning Incorporated, Lowell, MA). The chamber was incubated at 37°C at least 1 hour. The cells transfected with miRNA inhibitors were collected and resuspended in serum-free RPMI media at a density of 5 × 10^4^ cells/ml. The matrigel was gently washed with warmed serum free-RPMI media and 100 μl of the cell suspension was added onto the matrigel. The lower chamber of the transwell was filled with 650 μl RPMI media (10% FBS) and the plate was incubated at 37°C for 48 hours. The number of invaded cells was calculated as previously described [21].

### Small interference RNA (siRNA) transfection

To silence RBPMS, RCBTB1 and ZNF608, two siRNA for each target (Supplementary Table 2) and a non-silencing negative control siRNA (NC-siRNA) from Sigma-Aldrich, Inc. were used. A2780 (3.5 × 10^4^ cells/ml) were seeded into 10 cm Petri dishes. After 24 hours, siRNAs were mixed with the HiPerfect transfection reagent (Qiagen Inc., Valencia, CA) in a 1:2 ratio, and Opti-MEM I medium (serum/antibiotic-free). The transfection mix was incubated for 20 minutes at room temperature and then added to cells. Twenty-four hours later cells were collected for western blot analysis.

### Cell viability

A2780 cells (3.0 × 10^4^ cells/ml) were seeded into 96 well plates. Twenty-four hours later, siRNAs were added to the cells as described above. For combination treatment, eight hours after siRNA transfection, 1 μM of cisplatin (final concentration) was added to the cells. Seventy-two hours after siRNA transfection, the medium was removed and cell viability was measured using Alamar blue dye (Invitrogen, Carlsbad, CA, USA) as described previously [36].

### Computational analysis

The miRBase internet available miRNA-target prediction software (miRDB; microRNA.ORG, and Diana Lab) was used to identify the potential miR-21-3p target genes. The best 100 predicted miR-21-3p target from each of the software were selected as potential miR-21-3p target genes. MiR-21-3p potential target genes identified by the three softwares were chosen for further analysis. Based on the biological role and the complementarity between the miR-21-3p seed sequence to the 3′-UTR-binding sites in the target genes, 19 miR-21-3p targets were selected for further analysis.

### SYBR green-based RT-PCR for MiR-21-3p target gene detection

Total RNA was converted to cDNA using the iScript cDNA Synthesis Kit as per manufacturer's instructions (Bio-Rad, Hercules, CA, USA). Briefly, 4 μl of 5X iScript reaction mix, 1 μl of iScript reverse transcriptase, 1 μg of RNA template, and nuclease free water were mixed and incubated for 5 minutes at 25°C, 30 minutes at 42°C, 5 minutes at 85°C, and stored at 4°C. The PCR reaction containing 1 μl of cDNA, 1 μl of 20× Primer PCR assay, 10 μl of 2× SsoAdvanced SYBR Green supermix, and nuclease free water were subjected to the following conditions: Five minutes at 95°C followed by 40 cycles of 5 seconds at 95°C and 30 seconds at 60°C. Data was collected and analyzed using the StepOne software v2.1.

### Western blot analysis

Cells were collected, washed twice with Phosphate Buffer Saline (PBS), and stored at −80°C until processed. For total protein extraction, cells were lysed with a lysis buffer (150 mM NaCl, 1% Triton X-100, 0.4 mM NaF, 0.4 mM NaVO_4_, 25 mM Tris-HCl, pH 7.6 and 1× protease inhibitor) for 45 min on ice and then centrifuged for 10 min at 4°C. The supernatants were collected for further analysis. Protein concentration was determined using Bio-Rad Protein Reagents (Bio-Rad, Hercules, CA). Protein lysates (50 μg) were separated by SDS-PAGE, blotted onto membranes, and probed with the appropriate dilution of each primary antibody. Membranes were rinsed and incubated with a horseradish peroxidase-conjugated secondary antibody. Bound antibodies were detected using enhanced chemiluminescence (ECL) regents (GE Healthcare, Piscataway, NJ) and autoradiography using a FluorChem^™^ 8900 (Alpha Innotech Corporation, San Leandro, CA). The primary antibodies used were: anti-RBPMS (24 kDa), anti-RCBTB1 (58 kDa), anti-ZNF608 (162 kDa) (Novus Biologicals, Littleton, CO), and anti-β-actin (42 kDa) (Sigma, St. Louis, MO). The secondary antibodies used were anti-mouse and anti-rabbit IgG horseradish peroxidase (HRP) (Cell Signaling, Beverly, MA).

### Luciferase assays

For luciferase reporter assays, A2780 pre-mir-21 cells (3.5 × 10^4^ cells/ml) were transiently transfected with 1.5 μg of a dual Firefly/Renilla luciferase reporter mammalian expression vectors (pEZX-MT06; GeneCopoeia, Rockville, MD). The vectors included the 3′-UTR of RBPMS (Catalog # HmiT067170-MT01), RCBTB1 (Catalog # HmiT014179-MT01), ZNF608 (Catalog # HmiT015660-MT01), and PDCD4 (Catalog # HmiT007623-MT01). Each vector was mixed with Lipofectamine 2000 in a 1:1 ratio and Opti-MEM I. Eight hours post-transfection, the medium was replaced with fresh RPMI-1640 medium (with 10% FBS and 0.1% penicillin/streptomycin) and forty-eight hours post-transfection, the luciferase activity was measured. In cotransfection experiments, miR-21-3p-Inh or NC-miR-Ih were transfected in A2780-pre-mir-21 cells, and eight hours later the cells were transfected with the 3′-UTR vectors. Luciferase activity was detected in a Glomax 20/20 luminometer using a Dual-Luciferase Reporter Assay System (Promega, Madison, WI) following the manufacture's protocol. The relative luciferase activity was calculated as the ratio of firefly luciferase for each target relative to the firefly luciferase activity of the empty 3′-UTR vector. In cotransfection experiments, the luciferase activity was calculated relative to the NC-miR-Inh.

### Immunohistochemistry

Five formalin-fixed paraffin-embedded (FFPE) serous papillary ovarian cancer tissues (ages 38–76, mean 58 ± 11.63) and 5 normal ovary samples (ages 43–73, mean 51.89 ± 8.95) were kindly provided by the Department of Pathology at the University of Puerto Rico Medical Sciences Campus (UPR-MSC). This study was approved by the UPR-RCM IRB. A representative hematoxylin and eosin (H&E) stained slide from each paraffin block was reviewed by a pathologist to delineate the tumor areas and corroborate the tumor grade. Consecutive sections (5 μm thick) of each paraffin block were subjected to immunohistochemistry. Briefly, the slides were deparaffinized, re-hydrated, and them immersed in distilled water with 3% hydrogen peroxidase to suppress endogenous peroxidase activity. Antigen retrieval was performed by microwave treatment in antigen unmasking solution (Vector Laboratories, Inc, Burlingame, CA) for 15 minutes. Sections were incubated with RBPMS antibody (Abcam, Cambridge, MA) at a dilution of 1:100 in Dako antibody diluent (Dako North America Inc, Carpinteria, CA) overnight at 4°C. Subsequently, the Envision peroxidase-labeled polymer (goat anti-mouse; Dako North America Inc, Carpinteria, CA) was applied to the sections and signals were developed with diaminobenzidine (DAB) chromogen (Dako North America Inc, Carpinteria, CA). The immunoreactivity was estimated and graded by scoring the percentage of positive nuclear staining: score 1, negative; score 2, < 30%; score 3, 30 to 70%; and score 4, > 70%. The staining intensities (score 1: negative/weak staining intensity, score 2: intermediate staining intensity, and score 3: strong staining intensity, compared to the strongly stained tissue as the control.

### Statistical analysis

All experiments were performed at least in triplicate. Graphs were generated with the GRAPH PAD Prism 5 software (GraphPad Software, Inc., La Jolla, CA). Statistical analysis was performed using Student's *t*-test. *P*-values < 0.05 for a two-sided test were considered statistically significant.

## SUPPLEMENTARY MATERIALS FIGURES AND TABLES


