# Supplementary material for: Targeting miR-21-3p inhibits proliferation and invasion of ovarian cancer cells
Source: Oncotarget. 2016 May 7;7(24):36321–37. doi: 10.18632/oncotarget.9216 (PMC5095003; doi:10.18632/oncotarget.9216)
Supplement: Supplementary file 1 [file oncotarget-07-36321-s001.pdf]

Targeting miR-21-3p inhibits proliferation and invasion of ovarian cancer cells

Supplementary Materials

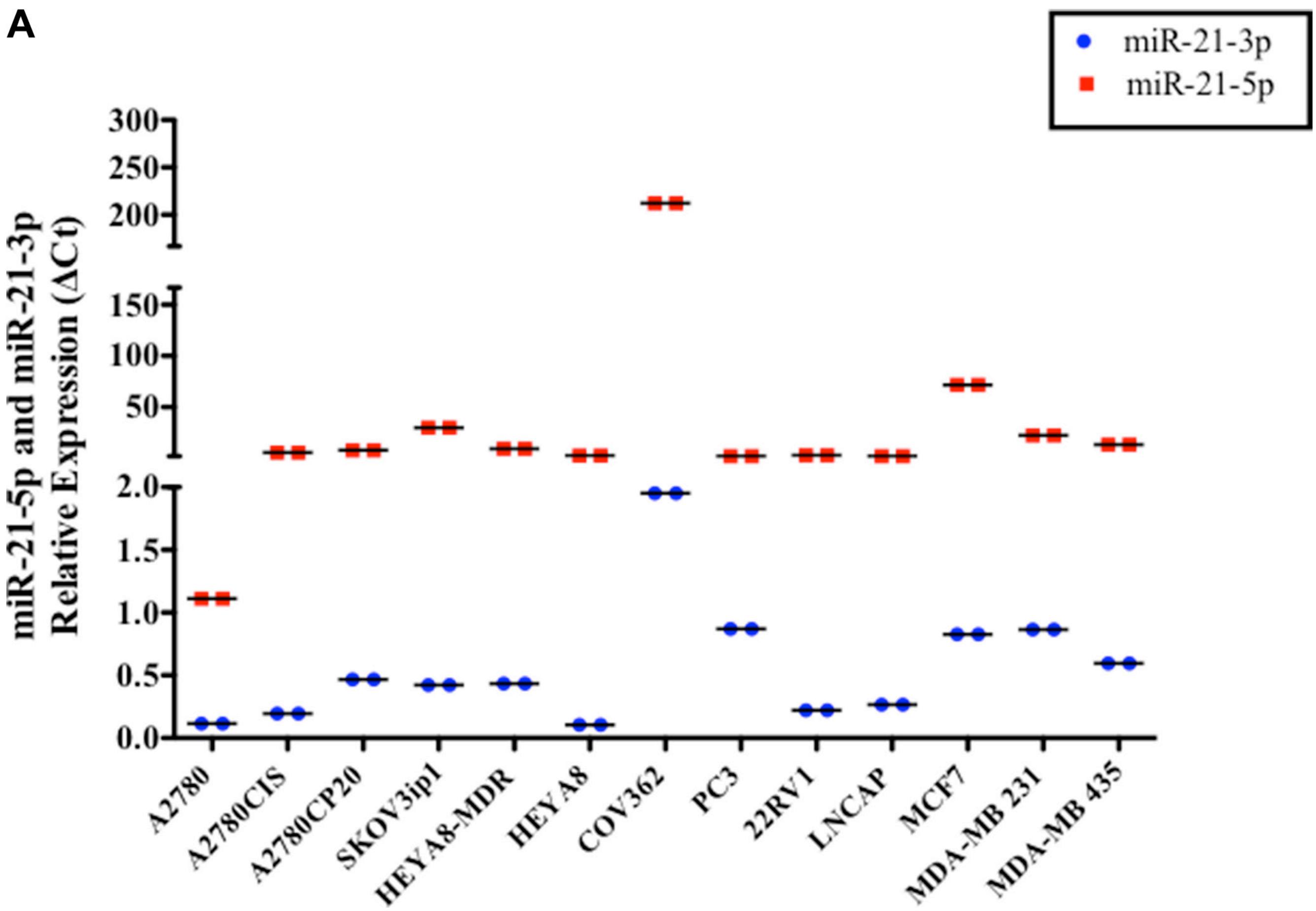

Supplementary Figure S1: MiR-21-5p and miR-21-3p ΔCt values in a panel of human cancer cells. (**A**) TaqMan-based real-time PCR analysis was performed as described in the "Materials and Methods" section. The delta threshold cycles (ΔCt) values were calculated as the *Ct* value of the miR-21-3p or miR-21-5p minus the *Ct* value of the internal standard (U44) for each cell line. Experiments were performed in triplicates.

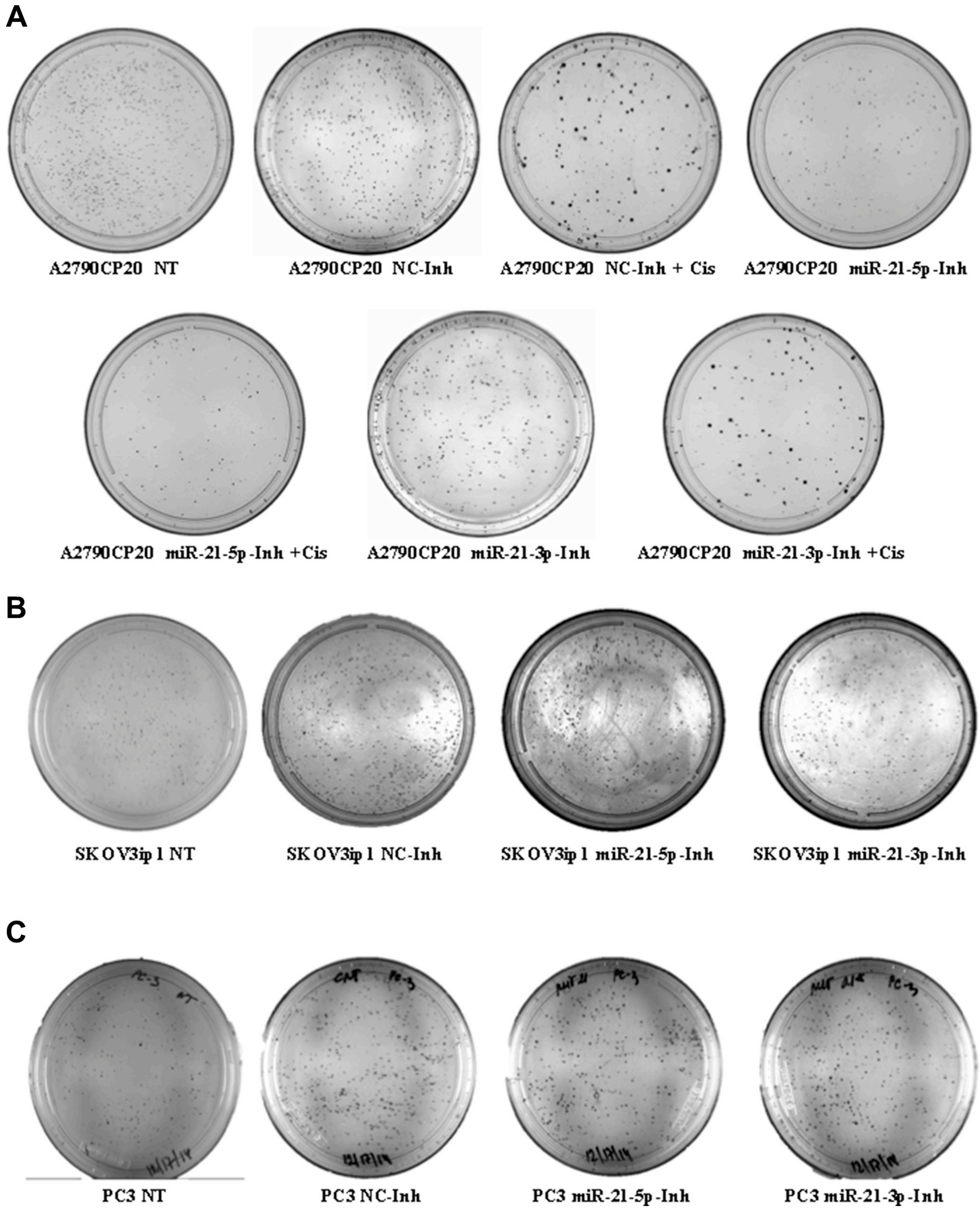

Supplementary Figure S2: Colony formation assays. A2780CP20, SKOV3ip1 and PC3 cells were transiently transfected with 50 nM (final concentration) of miR-21-5p-inh, miR-21-3p-inh or microRNA negative control inhibitor (NC-Inh) as described in the "Materials and Methods" section. Representative pictures of the Petri dishes with colonies of (**A**) A2780CP20 (with and without 5 µM of cisplatin), (**B**) SKOV3ip1 (**C**) and PC3 cells after transfection with NC, miR-21-5p and miR-21-3p inhibitors. Experiments were performed in triplicates.

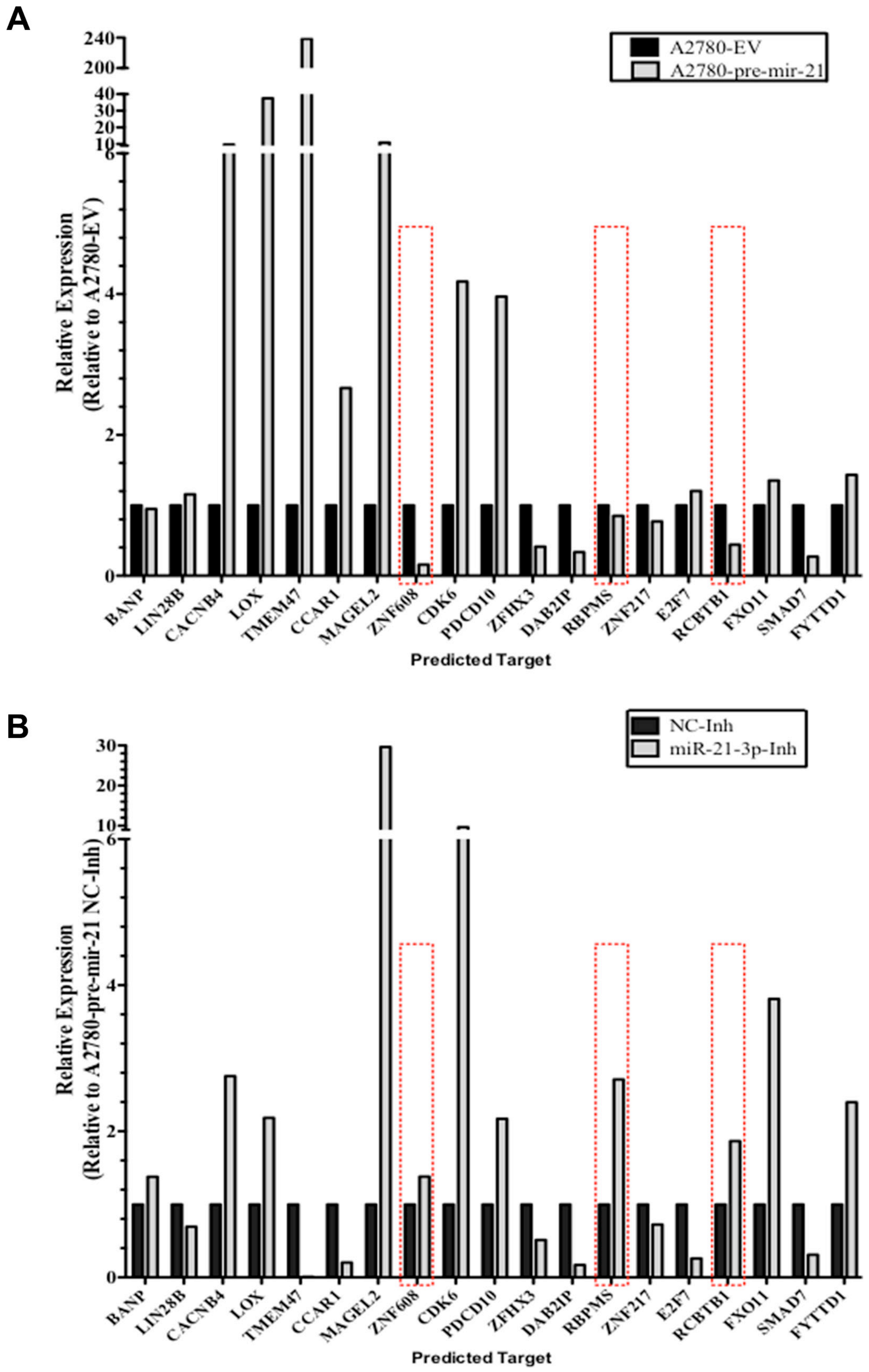

Supplementary Figure S3: Identification of the miR-21-3p targets by SYBR-Green RT-PCR. (**A**) SYBR-I-based real time-PCR for target validation was performed as described in the "Materials and Methods" section. Overexpression of pre-mir-21 in A2780 cells decreased the expression of ZNF608, ZFHX3, DAB21IP, RBPMS, RCBTB1, ZNF217 and SMAD7 genes. (**B**) Targeting of miR-21-3p in A2780 pre-mir-21 cells increased the expression of MAGEL2, ZNF608, RBPMS, RCBTB1, FBXO11 and FYTTD1.

A

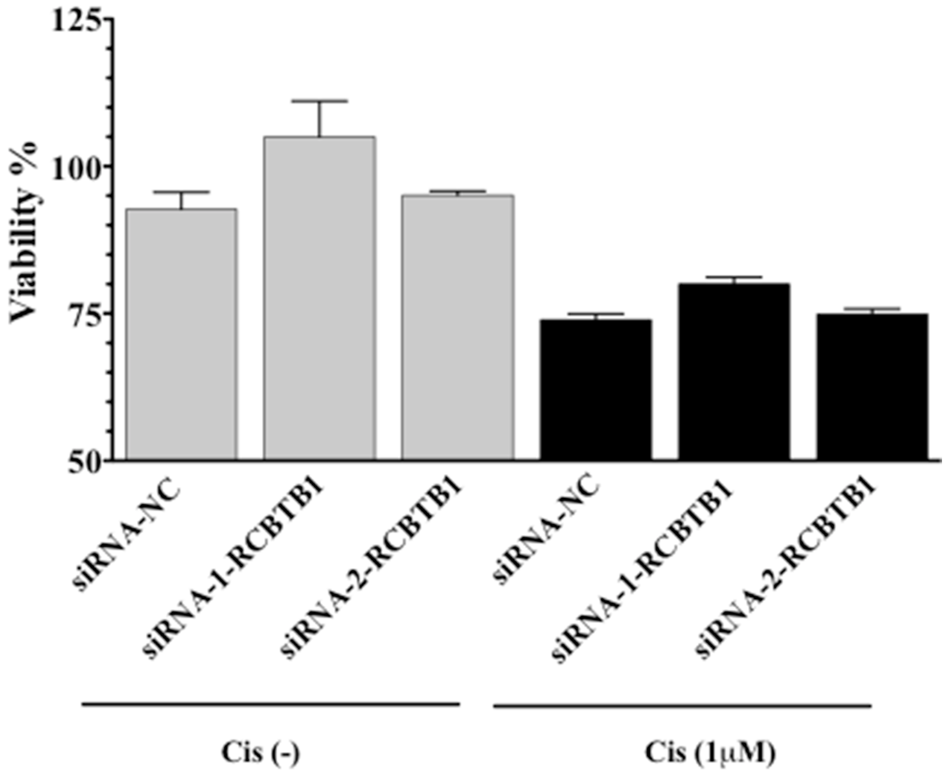

B

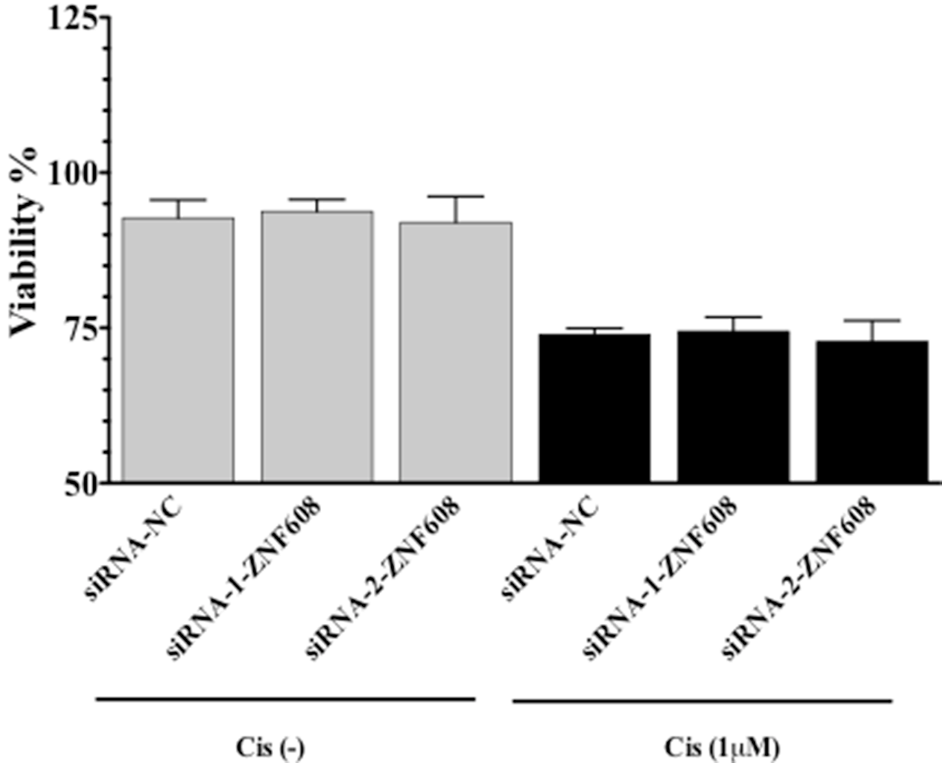

Supplementary Figure S4: SiRNA-mediated silencing of RCBTB1 and ZNF608 in A2780 cells. A2780 cells were transfected with two different siRNAs for (**A**) RCBTB1: siRNA-1-RCBTB1-1 or siRNA-2-RCBTB1, and (**B**) ZNF608: siRNA-1-ZNF608 or siRNA-2-ZNF608. SiRNA-transfected cells where exposed to 1 μM (final concentration) cisplatin. Forty-eight hours later cell viability was assessed as described in the "Materials and Methods" section. Experiments were performed at least in triplicates. Student t test was used for statistical analysis. Columns represent the means ± SEM.

Supplementary Table S1: MiRNA recognition sites in the 3-UTR of each of the 19 miR-21-3p target genes

Gene Symbol	Seed locations in the mRNA	Seed match type
BANP	308<br>521	7mer-A1<br>6mer
LIN28B	4071<br>4217	7mer-A1<br>7mer-A1
CACNB4	850<br>6176	6mer<br>7mer-A1
LOX	25<br>501	8mer<br>7mer-A1**
TMEM47	1056	7mer-A1
CCAR1	12	7mer-A1
MAGEL2	64<br>149	8mer<br>7mer-A1
ZNF608	60	7mer-A1
CDK6	1302<br>2088<br>7656<br>9786<br>9940	8mer<br>6mer<br>6mer<br>7mer-A1<br>7mer-A1**
PDCD10	285	7mer-A1
ZFHX3	836<br>2976<br>4010	7mer-A1**<br>6mer<br>7mer-A1**
DAB2IP	1055<br>1891	7mer-A1**<br>7mer-A1
RBPMS	1162<br>1648<br>1812<br>2143	7mer-A1<br>8mer<br>8mer<br>7mer-A1
ZNF217	2191	7mer-A1**
E2F7	457<br>2026	7mer-A1<br>6mer
RCBTB1	757<br>1107<br>1636	7mer-A1<br>7mer-A1<br>7mer-A1
FBX011	183<br>274	6mer<br>8mer
SMAD7	1416<br>1431	7mer-A1**<br>6mer
FYTTD1	625<br>1377<br>1857<br>2249	7mer-A1<br>6mer<br>6mer<br>7mer-A1

** Seed match type 7mer-A1, but it has a wobble pairing in the seed 8.

Supplementary Table S2: Recognition sites of the siRNA used for gene silencing

Accession Number	Gene Symbol	siRNA-1 Sequence (5′-3′)	siRNA-2 Sequence (5′-3′)
NM_001008710	RBPMS	GGGCTATGAGGGTTCTCTT	GCTATGAGGGTTCTCTTAT
NM_018191.3	RCBTB1	GTACCAATCTCTTGATCAA	CTATAGGTCTTCTGGATTT
NM_020747.2	ZNF608	GAAAGAGACGGGAAGCCCA	CCTTGTATGGGATTCCCGA
